# Doctors' Disabilities

**Published:** 1889-03-16

**Authors:** 


### The Hospital

A Weekly Institutional Journal of

**Science, Medicine, Nursing, and Philanthropy.**

*For Hospitals, Asylums, Medical Practitioners, Students, Nurses, Families, Parishes Congregations, and the Charitable Public.*

Vol. V. No. 129.] [March 16, 1889.

### Doctors' Disabilities.

The general community has a direct interest in putting every man into his proper place, and appraising each at his just value in the commonwealth. Mr. Herbert Spencer and others have taught us to regard the social system as a living organism, and to study it dispassionately and intelligently, exactly as the biologist studies a wheel animalcule, a seal, or a man. Such a method of study in nowise alters the facts of history or of political economy; but it enables us to see them from a new standpoint; to cast upon them as it were the light of a new illuminator; to take a juster measure of them than before, and to convince ourselves more clearly of their excellences and of their defects.

At different periods of life the human creature displays totally different sets of characteristics. In youth, for example, self-indulgence and self-will are prominently in evidence; and the phrase that "a man must sow his wild oats," expresses a very common belief in the overmastering nature of youthful passions. In early manhood the sense of all-round responsibility is perhaps the most marked circumstance of daily experience. The young man takes things very seriously, as if he were oppressed with the consciousness that mistakes are easily made, and that success is very difficult to attain. At a still later stage the critical faculty becomes more developed, and progress is sought rather by the exercise of judgment and the careful adaptation of means to ends than by the method of "storming" and the use of the bayonet. All these facts have their analogues and resemblances in the life of the social system.

In this country, which no one would now call anything but an "old country," we have reached the stage of criticism and even-mindedness. Although there are among us many who counsel that "raw haste" which, as Tennyson says, is "half-sister to delay," yet the majority of men of light and leading have faith in the doctrine of "Festina Lente." The critical faculty, under these circumstances, is developing day by day. Perhaps the most competent critics of the present time are the men of science, among whom may be included intelligent medical men. Their special training is almost entirely observational and critical. They learn, above all things, the methods of seeing clearly, judging according to the facts of the case, and classifying correctly. Passion and prejudice are held by them as enemies, and the pure light of reason is their torch and sun. It would seem natural that men of such training and habits should exercise much authority and influence among their less critical and judicial fellows; that not only would their judgment be supreme in their own particular sphere, but that they should even be asked to adjudicate upon questions of science and fact outside that sphere. Instead of this, we find that scientific men, and particularly the medical portion of them, are by common consent placed outside the march of the world's every-day business, and relegated to the hospital, the consulting-room, and the bedside almost exclusively.

That hotel-keeper would not be considered a good man of business who should make his high-salaried French cook devote his whole time to knife-cleaning and pan-scouring; the squire who should put his best hunter to the plough or the hay-cart would be regarded as anything but a philosopher. He only is fit to be the possessor of good instruments who knows how to put them to the best possible use. In the present condition of its public intelligence, Great Britain is entirely unworthy of its noble army of scientific men. There is not one of them of whom it makes the best use, whilst many of them it never uses at all. What, for example, would be more scientific and rational than that medical men should be extensively charged with the oversight of the youth of the nation? During the period of growth and development the body is surely of as great importance as the mind! Yet children, boys and girls, and young people are handed over body and soul to the exclusive charge of the schoolmaster. The results of this course on a large scale it would be impossible to compute. It is certain that mischiefs and miseries of all kinds spring from it, and that many premature deaths are due to it and to it only. The positive injury that is done is, however, only a part of the evil arising from the exclusion of the doctor from the domain of the training of youth. The absence of robustness, of physical energy, of lightness of spirits, and of abounding capacity of all kinds, may be traced to that want of rational physical management which an intelligent physician with full powers could so well supply. The plain truth is that the unscientific public has not the wit to perceive the doctor's value in this respect. It will employ him to patch up its broken-down property, but it will not set him to work to help to build up its new human buildings to a higher and more enduring perfection. It will permit him to follow in the wake of the schoolmaster, in order to take care of the wounded and to certify the dead; but it will not have him to act as a check upon the schoolmaster, or, when occasion requires, as a leader and guide. Both the schoolmaster and the public resent ideas of this kind, and that to their own very great injury and loss. But this one thing is certain, that if the teachings of scientific doctors continue to be as much ignored as the doctors themselves are, it is not the doctors who will suffer, but the whole population. The British people must either learn to live according to physiological principles, or they will not continue to live at all as a free nation. Nothing whatever, neither mental training nor moral excellence, can compensate for the want of a stalwart physique.

If the guardians of youth are so wanting in scientific intelligence as to despise the teachers of the gospel of physiology, lawyers of every grade go far beyond schoolmasters in their ignorance and contempt. The British lawyer is a curious creature. His business is usually to deal with facts. His training is for the most part confined to books. Even his books he often makes a poor use of, being generally far more concerned in the quoting of a precedent than in the maintaining of a principle. The sorriest spectacle that ever lawyers present is when they are gravely trying their wits on a medical case. They ask the most absurd questions in the gravest of legal manners, and listen to the most scientific or the most inconsequent answers with equal solemnity and equal incomprehension. Not only are pleaders as a rule utterly ignorant of the very elements of medical and physiological science, but even judges themselves, who have to direct juries, are for the most part in exactly the same condition of scientific darkness. A doctor or a surgeon of the profoundest learning, the most scrupulous honesty, and the most proved ability in science and practice, may be pitted against an ignorant and unconscientious blockhead, and the evidence of the one shall, in the judges' eyes, be of exactly the same value as that of the other. To ask an accomplished physician or surgeon to submit to cross-examination at the hands of a barrister who knows nothing of science, is as great an insult and as futile a proceeding as it would be to ask Sir Frederick Leighton to be examined in painting by the parish fiddler. Yet this is constantly done, to the gross miscarriage of justice and the scandal of public morality. In all trials involving medical circumstances one or more doctors should be on the Bench, either as sole judges or as professional assessors. Only thus can anything like justice be done. This is not a question for doctors exclusively, or even chiefly. The public suffer when justice miscarries, and the law, with its administrators, is dishonoured.

It would be easy to give many other illustrations of the way in which medical science is misunderstood and despised. The folly of the public in so despising it is great beyond expression. It cannot require a philosopher to see that he who shuts himself up in a room with a farthing candle, when he might be abroad in the light of the sun, is not far removed from a fool. How much wiser are those people who, when the sun of science is shining brightly and clearly, refuse absolutely to walk in its light in many of the most important concerns of life? Medical men are a timid race, and do not strive to remove those disabilities which keep them in a secondary or still lower position, when they ought to be first. But the world ought to both know and insist that when a round hole is to be filled a round man shall be put into it, and when scientific and medical knowledge are essential to the wellbeing of the people and the just administration of the law, such knowledge shall be made use of at whatever cost. A rational public will recognize the value of all its servants, and will put every one of them to the particular use for which he is most fitted by nature and training.

